# Omnipod 5 outcomes comparing Dexcom G6 and Freestyle Libre 2 plus users in adults with type 1 diabetes

**DOI:** 10.1111/dme.15465

**Published:** 2024-11-04

**Authors:** Roland H. Stimson, Anna R. Dover, Marcus J. Lyall, Catriona J. Kyle, Rohana J. Wright, Gayle McRobert, Mark W. J. Strachan, Fraser W. Gibb

**Affiliations:** ^1^ Edinburgh Centre for Endocrinology and Diabetes Edinburgh United Kingdom of Great Britain and Northern Ireland

Omnipod 5 (OP5) is a tubeless automated insulin delivery (AID) system that was, until recently, only compatible with Dexcom G6 sensors (G6). Currently, all published evidence attesting to the efficacy of OP5 relates specifically to use with G6 sensors.^1^, ^2^ In mid‐2024, OP5 compatibility with Freestyle Libre 2 Plus CGM (L2+) was launched in the United Kingdom. This study aimed to compare early glycaemic outcomes, and time spent in AID mode, between those using G6 and L2+ sensors.

This was a retrospective analysis of 77 adults (45 G6 and 32 L2+) with type 1 diabetes at a single centre in the UK. Baseline CGM data were collected in the 28 days prior to converting from Omnipod DASH (standalone CSII) to OP5 and compared with data from the first 28 days of AID use. CGM data were obtained from LibreView and Glooko. Clinical and demographic data were obtained from SCI Diabetes (national diabetes register). As a service evaluation of routinely collected data, this project did not require ethical approval. Paired data were compared with Wilcoxon‐signed rank tests and unpaired data with Wilcoxon rank‐sum test. Correlations were assessed by the Spearman correlation coefficient. Categorical data were compared by chi‐squared test. Logistic regression analysis assessed independent predictors of reduction in TBR and improvement in TIR (defined as >10%). *p* values <0.05 were considered statistically significant. Statistical analyses were performed using R Studio (version 2023.12.1).

Forty‐nine (64%) were female. Median age was 43 years (IQR 32–56) and diabetes duration was 23 years (14–33). Age (*p* = 0.872), duration (*p* = 0.371), sex (*p* = 0.947), socio‐economic deprivation (*p* = 0.912) and BMI (*p* = 0.161) did not differ between L2+ and G6 users at baseline. Baseline CGM metrics were not significantly different between G6 and L2+ users (all *p* > 0.05).

Time in automode did not differ between groups (G6: 99% [96–100] vs. L2+: 99 [98–100], *p* = 0.551) and this was also true of time in limited automode (G6: 2% [2–3] vs. L2+: 2 [1–3], *p* = 0.826). Changes in glucose metrics from baseline are presented in Table 1. Correlation (R) between baseline TIR and change in TIR was −0.91 for L2+ and −0.56 for G6 (both *p* < 0.001). Logistic regression analysis identified only baseline TIR (OR 0.93 per % [95% CI 0.88–0.97], *p* < 0.001) but not sensor type (*p* = 0.723) as an independent predictor of >10% improvement in TIR. Any fall in TBR was independently associated with baseline TBR (OR 3.4 per % [95% CI 2.0–7.1], *p* < 0.01) and also the use of G6 sensors (OR 5.8 [95% CI 1.4–34], *p* = 0.027).

**TABLE 1:** Comparison of CGM metrics at baseline and after 28 days in both Dexcom G6 and Libre 2 Plus users and comparison of differences at 28 days between Dexcom G6 users and Libre 2 Plus users.

	Baseline Dexcom G6 cohort	Post Dexcom G6 OP5 use	*p*	Baseline Libre 2+ cohort	Post Libre 2+ OP5 use	*p*	Δ after OP5 use (Dexcom G6)	Δ after OP5 use (Libre 2+)	*p*
Average glucose (mM)	11.3 (9.5–12.2)	9.5 (8.7–10.3)	<0.001	9.9 (8.9–11.5)	8.9 (8.1–9.4)	<0.001	−1.3 (−2.0 to −0.7)	−1.6 (−2.2 to 0.7)	0.768
GMI (mmol/mol)	66 (57–70)	57 (54–61)	<0.001	60 (55–67)	55 (55–57)	<0.001	−6 (−9 to −3)	−7 (−10 to −4)	0.816
CV glucose (%)	36.4 (33.8–41.3)	31.9 (29.5–34.9)	<0.001	36.4 (33.6–38.8)	34.9 (32.0–38.0)	0.197	−4 0.0 (−7.7 to −1.2)	−1.6 (−4.3 to 1.8)	0.008
Glucose <3 mM (%)	0.2 (0.6)	0.2 (0.3)	0.152	0.3 (0.6)	0.2 (0.3)	0.304	−0.1 (0.5)	−0.1 (0.7)	0.872
Glucose 3–3.8 mM (%)	1 (1–3)	1 (0–1)	<0.001	1 (0–2)	2 (1–3)	0.291	−1 (−1 to 0)	1 (−1 to 1)	0.003
TBR (%)	1 (1–3)	1 (0–1)	<0.001	1 (0–4)	2 (1–3)	0.558	−1 (−1 to 0)	1 (−1 to 1)	0.002
TIR (%)	42 (33–58)	61 (54–75)	<0.001	55 (36–65)	70 (64–75)	<0.001	17 (12 to 23)	19 (11 to 23)	0.788
Glucose 10.1–13.9 mM (%)	29 (26–33)	26 (19–31)	0.004	28 (22–33)	22 (17–26)	0.002	−4 (−8 to 1)	−5 (−13 to −1)	0.191
Glucose >13.9 mM (%)	27 (12–24)	11 (4–14)	<0.001	13 (8–27)	6 (3–10)	<0.001	−14 (−18 to −5)	−9 (−17 to −3)	0.238
TAR (%)	57 (38–66)	37 (24–46)	<0.001	44 (33–64)	30 (22–34)	<0.001	−17 (−23 to −11)	−19 (−24 to −9)	0.528

*Note*: All data median (IQR) except glucose <3 mM which is presented as mean (SD).

There do not appear to be any important differences in time spent in automode between G6 and L2+. Similarly, early CGM outcomes are comparable between the two CGM options with respect to average glucose, TIR and high glucose metrics. Although the follow‐up duration of 28 days is relatively short, TIR outcomes are broadly similar to our recently published 1‐year follow‐up data,^3^ suggesting early changes are a durable indicator of longer‐term outcomes. Significant improvement in TBR was confined to G6 users but it is debatable whether comparison of such small differences between two CGM systems is clinically meaningful; especially in a population with such low baseline TBR. There is a dearth of published data directly comparing how accurately L2+ and G6 identify glucose levels in the hypoglycaemic range. A small comparison of Freestyle Libre versus Dexcom G6, in non‐diabetic adults (30 days of CGM use), reported a TBR of 9.1% using Libre results versus 1.4% using G6.^4^ These observations suggest we should exercise caution in inferring too much from small differences in TBR between two different glucose sensors. Reduction in CV glucose was greater in the G6 cohort, which could represent differences in the sensor‐algorithm interaction but may also reflect baseline differences in TAR. The question of whether one CGM system truly results in superior reduction in TBR and CV glucose will only be resolved by a study with concurrent use of both sensors. These early data offer reassurance that sensor choice with OP5 does not result in clinically meaningful differences in automode performance or CGM metric outcomes.

## CONFLICT OF INTEREST STATEMENT

FWG has received speaker fees from Abbott, Dexcom and Insulet. ARD has received speaker fees from Abbott.

## FUNDING INFORMATION

No funding was received.
